# Supplementary material for: Bayesian Unidimensional Scaling for visualizing uncertainty in high dimensional datasets with latent ordering of observations
Source: BMC Bioinformatics. 2017 Sep 13;18(Suppl 10):394. doi: 10.1186/s12859-017-1790-x (PMC5606221; doi:10.1186/s12859-017-1790-x)
Supplement: Additional file 1 — Supplementary figures showing comparison between heatmaps reordered with BUDS and NeatMaps. Figure S1. Comparison of BUDS and NeatMap for matrix ordering applied to three biological datasets. The heatmaps are shown for 500 randomly selected features (the same for BUDS and Neatmap). A default color scheme setting was for NeatMap heatmaps. BUDS ordering gives a much clearer visualization of the continuous patterns present in the data. Figure S2. Comparison of BUDS and NeatMap for matrix ordering applied to three biological datasets. The heatmaps are shown for 500 randomly selected features (the same for BUDS and Neatmap). A viridis color scheme setting was for NeatMap heatmaps. (PDF 321 kb) [file 12859_2017_1790_MOESM1_ESM.pdf]

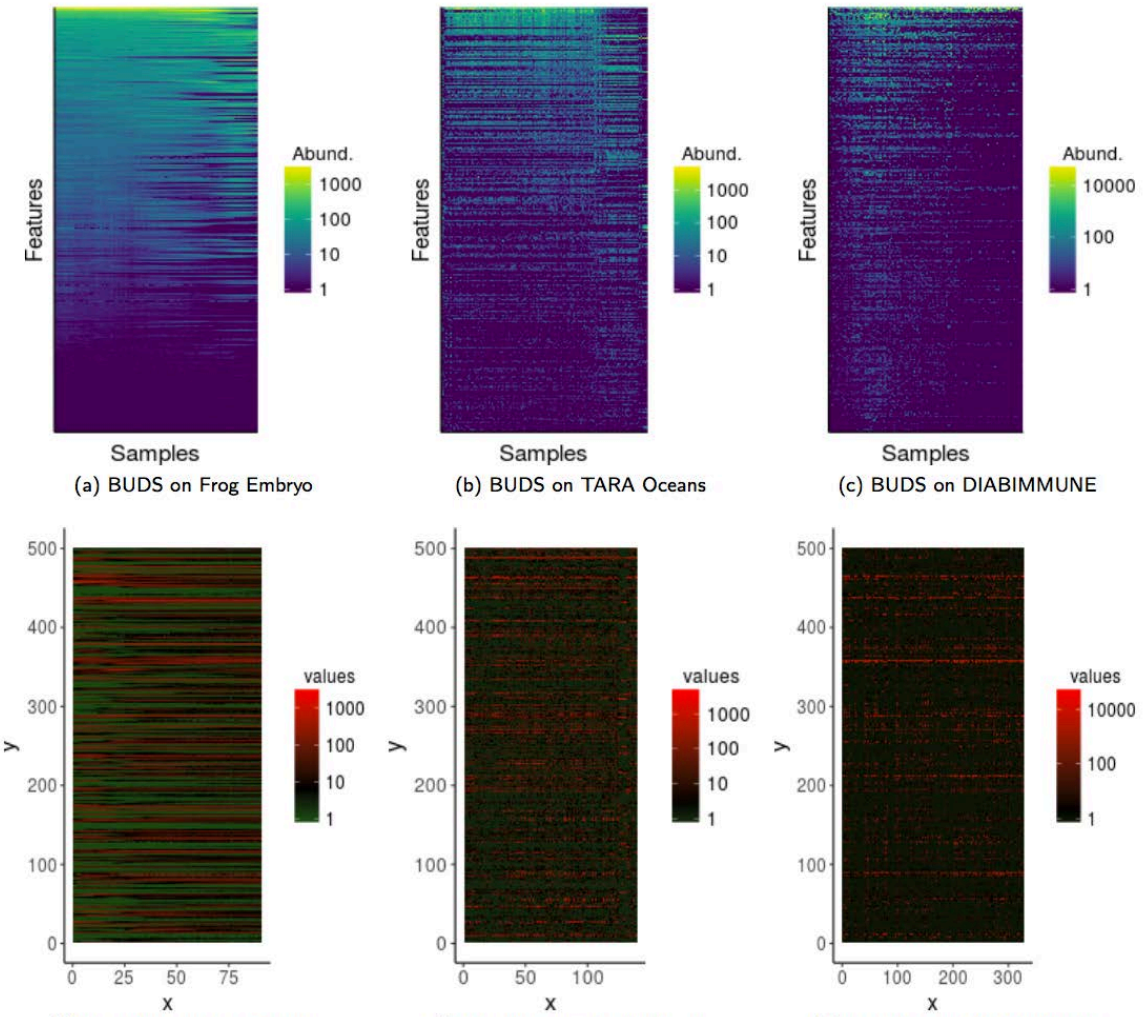

(a) BUDS on Frog Embryo
(b) BUDS on TARA Oceans
(c) BUDS on DIABIMMUNE
(d) NeatMap on Frog Embryo
(e) NeatMap on TARA Oceans
(f) NeatMap on DIABIMMUNE

Figure S1 Comparison of BUDS and NeatMap for matrix ordering applied to three biological datasets. The heatmaps are shown for 500 randomly selected features (the same for BUDS and Neatmap). A default color scheme setting was for NeatMap heatmaps. BUDS ordering gives a much clearer visualization of the continuous patterns present in the data.

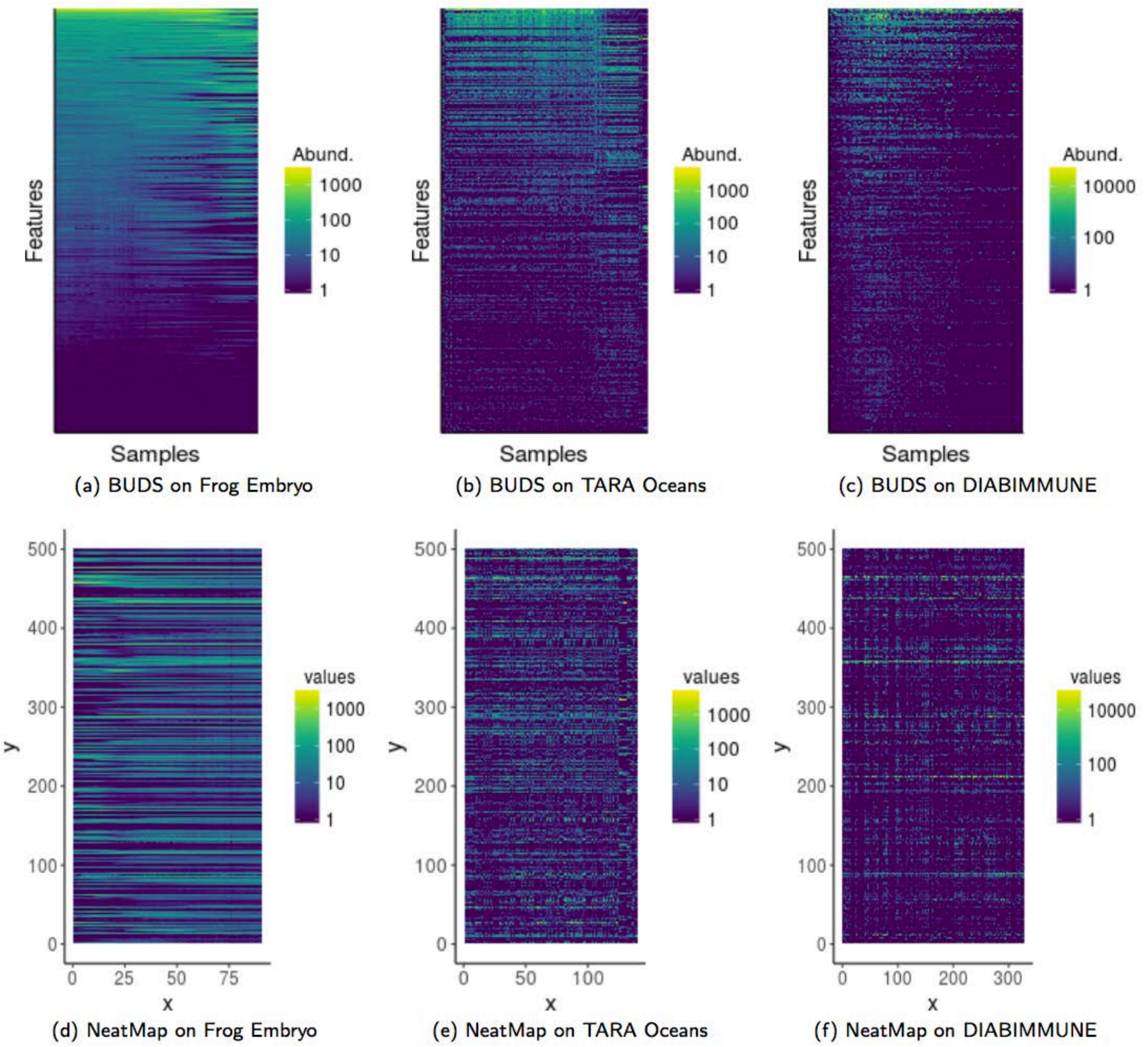

(a) BUDS on Frog Embryo (b) BUDS on TARA Oceans (c) BUDS on DIABIMMUNE

(d) NeatMap on Frog Embryo (e) NeatMap on TARA Oceans (f) NeatMap on DIABIMMUNE

Figure S2 Comparison of BUDS and NeatMap for matrix ordering applied to three biological datasets. The heatmaps are shown for 500 randomly selected features (the same for BUDS and Neatmap). A viridis color scheme setting was for NeatMap heatmaps. BUDS ordering gives a much clearer visualization of the continuous patterns present in the data.
